# The effect of a home exercise program on visio-vestibular function in concussed pediatric patients

**DOI:** 10.3389/fspor.2023.1064771

**Published:** 2023-03-03

**Authors:** Patricia R. Roby, Olivia E. Podolak, Matthew Grady, Kristy B. Arbogast, Christina L. Master

**Affiliations:** ^1^ Center for Injury Research and Prevention, The Children’s Hospital of Philadelphia, Philadelphia, PA, United States; ^2^ Perelman School of Medicine, University of Pennsylvania, Philadelphia, PA, United States; ^3^ Sports Medicine Performance Center, The Children’s Hospital of Philadelphia, Philadelphia, PA, United States; ^4^ Division of Emergency Medicine, The Children’s Hospital of Philadelphia, Philadelphia, PA, United States

**Keywords:** vision, vestibular, therapy, adolescent, symptom

## Abstract

**Background:**

A visio-vestibular home exercise program (VV-HEP) can provide an equitable and cost-effective method for therapy targeted towards visio-vestibular deficits that are common following concussion. The effects of a VV-HEP on improving concussion symptoms and visio-vestibular function are unclear.

**Purpose:**

Determine the effect of VV-HEP on symptoms and visio-vestibular function in concussed pediatric patients.

**Methods:**

This study included 527 patients [294 female (55.8%); age = 14.4 ± 2.1 years] reporting to a specialty care concussion center within 28 days of injury and for a first follow-up within 60 days of injury. Patients completed the Post-Concussion Symptom Inventory (PCSI) and Visio-Vestibular Examination (VVE). Patients were prescribed the VV-HEP at initial visit, with exercises including saccades, gaze stability, convergence, and balance, and instructed to complete these 1–2 times/day. At follow-up, patients self-reported their VV-HEP progress as (1) has not done, (2) in progress, or (3) completed. Primary outcomes included VV-HEP progress at follow-up, PCSI endorsement and severity, VVE subtests (normal/abnormal), and total VVE score (abnormal = 2 + abnormal subtests). Kruskal-Wallis tests and chi-square were used to determine if concussion symptoms or the proportion of abnormal VVE outcomes, respectively, were associated with VV-HEP status. *Post-hoc* pairwise comparisons with Bonferonni corrections were used to determine concussion symptom (*α* = 0.017 *a priori*) and VVE (*α* = 0.005 *a priori*) differences in VV-HEP status.

**Results:**

At follow-up, patients who had completed the VV-HEP reported lower symptom endorsement (median = 1, IQR = 0–3) and lower symptom severity (median = 1, IQR = 0–4) relative to patients who had not started the VV-HEP (endorsement median = 7, IQR = 1–13, *p* < 0.0001; severity median = 15.5, IQR = 2–32.5, *p* < 0.0001) and those in progress (endorsement median = 8, IQR = 3–14, *p* < 0.0001; severity median = 15, IQR = 4–30, *p* < 0.0001). A lower proportion of patients who completed the VV-HEP reported with abnormal vestibular-ocular reflex (22.2%), tandem gait (0%), and total VVE score (22.2%) relative to those who had not started or those in progress (*p* < 0.005).

**Conclusion:**

Our findings indicate that patients who completed the VV-HEP had lower symptom burden and improved visio-vestibular function relative to those who did not start or were in progress. This suggests that a VV-HEP can effectively reduce visio-vestibular dysfunction following concussion and may serve as a means to minimize inequities in access to care.

## Introduction

An estimated 1.1–1.9 million sport- and recreation-related concussions occur in patients 18 or younger annually (1), a majority of which enter the health care system through primary care physicians (2). Current concussion consensus statements recommend a multi-faceted approach to concussion management (3), particularly in pediatric patients due to an increased vulnerability to injury (4), longer average recovery (5), and the inclusion of additional stakeholders (i.e., school nurses, teachers, parents) (5) relative to adult patients. Additionally, active rehabilitation programs, including vision and vestibular therapies, are recommended for the treatment of adolescent concussion (3, 6–8); however, barriers to specialized concussion care, including financial barriers (9, 10), access to health care (11), or socioeconomic health disparities (12–14), prevent patients from accessing the relevant concussion care to optimize their recovery. There is a need to further explore cost-effective and equitable approaches to active concussion management in a pediatric population.

Visual and vestibular impairments are estimated to occur in up to 88% of adolescent concussion patients (15–17) and have been associated with prolonged recovery (17). In-office therapy targeting vision and vestibular function has demonstrated improved clinical outcomes, including overall symptom reduction (18), improved vestibulo/ocular-motor performance (19), and earlier medical clearance (20) in adults and adolescents. Additionally, early initiation (≤30 days) of vestibular physical therapy has been associated with earlier return-to-play and symptom resolution in pediatric and young adult patients (aged 5–23 years) (21). When examining at-home therapy strategies, Kontos et al. (22) reports encouraging preliminary findings that adolescent patients enrolled in a 4-week at-home vestibular rehabilitation program demonstrated improved vestibular-ocular reflex (VOR) compared to controls (22). Further, home exercise programs targeting vestibular/ocular-motor rehabilitation have been associated with fewer recovery days relative to prescribed physical therapy in pediatric patients (23), suggesting that home programs may be an effective strategy to improve visio-vestibular function. These previous studies were either limited by a small sample size or did not assess progress with the home program upon follow-up, making it critical to build on these preliminary findings to further investigate the effectiveness of such programs.

Our primary purpose was to investigate the effect of a visio-vestibular home exercise program (VV-HEP) on concussion symptom reporting during follow-up visits within 60 days of injury in pediatric patients. Our secondary purpose was to determine if a VV-HEP improved visio-vestibular function in the same cohort.

## Methods

**Figure 1:**
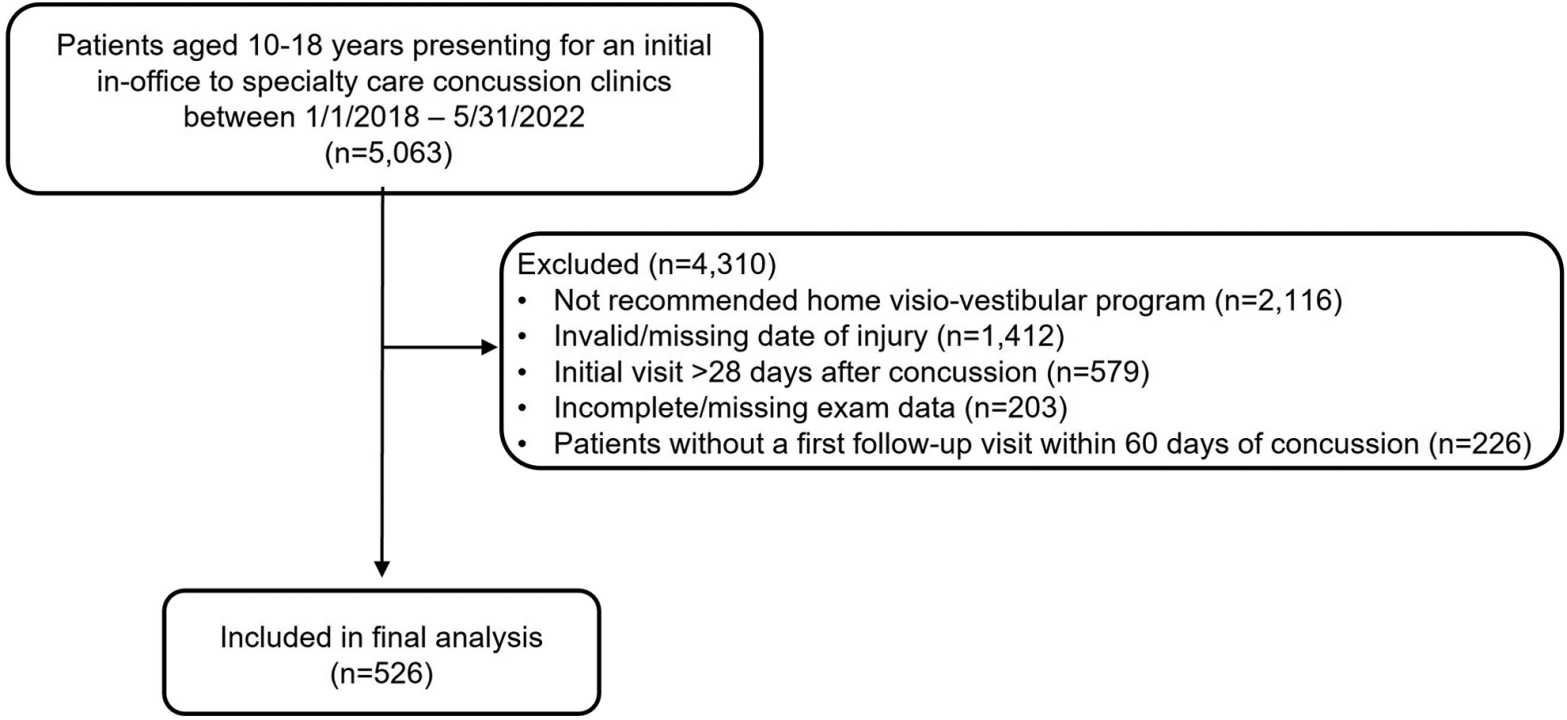
Consort diagram for patients included in final analysis.

Data were prospectively queried from the Minds Matter Concussion Registry using electronic health records (EHR) for patients seen for concussion within the Children's Hospital of Philadelphia (CHOP) pediatric network. For this analysis, the population was limited to patients aged 10–18 years old presenting for their initial visit to the specialty care concussion program as a part of our Sport Medicine and Performance Center within 28 days of injury (5) between January 1, 2018 and May 31, 2022. Patients were diagnosed by a physician trained in concussion using the definition of concussion set forth in the Consensus Statement on Concussion in Sport (3). Patients were included in the follow-up analysis if they were subsequently seen for a first follow-up within 60 days of injury, based on clinical need. The derivation of the study population is described Figure 1. The study was approved by the CHOP Institutional Review Board (IRB# 19-016019).

At each visit, concussion symptoms were assessed using the Post-Concussion Symptom Inventory (PCSI), which asks patients to self-report symptoms and symptom severity on a scale from 0 (not a problem) to 6 (severe problem). Patients completed an age-specific PCSI based on age (8–12 or 13–18 years), both of which demonstrate good test-retest reliability (8–12 years intraclass correlation coefficient = 0.89, 13–18 years intraclass correlation coefficient = 0.79) (24). Primary outcomes from the PCSI included total symptom endorsement and total symptom severity. Visio-vestibular function was assessed using a visio-vestibular examination (VVE), which is a battery of 9 subtests including smooth pursuit, horizontal/vertical saccades, horizontal/vertical VOR, near point of convergence (NPC), left and right monocular accommodation, and complex tandem gait (Supplementary Material; Video VVE demonstration: https://www.chop.edu/video/pediatric-exams-concussion-evaluation). To determine performance, the VVE considers presence of self-reported symptom provocation (including headache, dizziness, eye fatigue, eye pain, or nausea) (yes/no), physical signs (yes/no), and established repetition-based cut-offs allowing for increased concussion diagnosis sensitivity and a more comprehensive assessment of visio-vestibular function (25, 26). The VVE has been feasibly administered across different health care settings (27, 28), and shows fair to moderate agreement between providers and moderate to substantial agreement with the same provider (25). The primary outcomes from the VVE included subtest outcomes (normal vs. abnormal) and total VVE score (abnormal: ≥ 2 abnormal subtests) (28). The VV-HEP consisted of exercises targeting visual and vestibular function including saccades, VOR, convergence, and balance (Supplementary Material). Patients were instructed during their initial visit to complete exercises 1–2 times daily and were informed we would be following up with their progress at subsequent visits but were not otherwise given any reminders or incentive to comply with the VV-HEP.

Prior to the start of the clinical exam, patients completed a demographic questionnaire, including age and patient-reported medical history, and physicians used a standardized template within the EHR to document injury and self-reported concussion history details. During the exam, patients completed the VVE and PCSI as a part of a comprehensive concussion assessment. Following the exam, physicians indicated if the patient was prescribed a VV-HEP. At each clinical follow-up, patients repeated the VVE and PCSI and self-reported their progress on the VV-HEP to the provider, who recorded the response in the EHR from a drop-down list of options. Progress was categorized as “has not started,” “in progress,” or “has completed.” Responses recorded as having tried the HEP but stopped and patients who never tried the HEP were categorized as “has not started.” Responses recorded as “is doing the HEP” were categorized as “in progress.” Responses recorded as “has completed the HEP” were categorized as “completed.” Patients were excluded if they reported an invalid or missing date of injury [e.g., clinician or patient failed to document date, missing month, day, or year, documentation error (date of injury is date of birth or a date in the future)], if they were not prescribed the VV-HEP, or if they had incomplete VVE or PCSI.

Descriptive statistics were used to describe patient demographics, characterize concussion history and injury details, and self-reported progress with the VV-HEP. Our primary analysis compared symptom endorsement and symptom severity between patients who self-reported having not started, those who were in progress, and those who had completed the VV-HEP. Kruskal-Wallis tests were used to determine if self-reported progress with the VV-HEP (“had not done,” “in progress,” or “completed”) was associated with PCSI symptom endorsement or PCSI symptom severity. *Post-hoc* pairwise comparisons with Bonferroni adjustment (alpha = 0.017) were used to further delineate differences in symptom reporting based on VV-HEP status. Effects sizes for symptom reporting were calculated using eta squared (ƞ^2^). Our secondary analysis used chi-square tests to compare VVE abnormalities by self-reported progress with the VV-HEP. To adjust for 10 comparisons in our secondary analysis, Bonferroni adjusted alpha was set to 0.005 *a priori*. Effect sizes for self-reported progress with VV-HEP were calculated using Cramer's’ V. Data were analyzed using SAS statistical software, version 9.4 (SAS Institute Inc., Cary, NC).

## Results

A total of 527 patients were included in the final analysis [female = 294 (55.8%), age = 14.4 ± 2.1 years] (Table 1). The majority of patients reported with no prior concussion history (54.1%), with a sport-related mechanism for the current concussion (69.1%), and a median lifetime concussions of 1 (IQR = 1–2). Patients were seen for an initial visit an average of 11.1 ± 6.9 days after concussion and first follow-up an average of 30 ± 13.2 days after concussion. Patients had a median of 2 (IQR = 1–4) abnormal VVE subtests at initial visit and a median of 2 (IQR = 0–5) abnormal VVE subtests at first follow-up. At first follow-up, patients were seen an average of 19.0 ± 9.2 days after their initial visit and most patients self-reported being in progress with the VV-HEP (*n* = 412, 78.3%), followed by patients who had not started (*n* = 81, 15.3%), and patients who had “completed” the program (*n* = 27, 5.1%).

**Table 1:** Demographic information for patients at initial visit.

	*n* = 527
Sex, *n* (%)	
* Male*	233 (44.2%)
* Female*	294 (55.8%)
Age, years, mean (SD)	14.4 (2.1)
Race/Ethnicity, *n* (%)	
* Non-hispanic white*	383 (72.8%)
* Non-hispanic black*	49 (9.3%)
* Hispanic*	29 (5.5%)
* Non-hispanic Asian/Asian Pacific Islander/other/multiple race/unknown*	65 (12.4%)
Concussion history, *n* (%)	242 (45.9%)
Lifetime concussions, median (IQR)	1 (1–2)
Current concussion was sport-related, *n* (%)	364 (69.1%)
Days since injury, mean (SD)	11.1 (6.9)

When assessing concussion symptom endorsement and severity at initial visit, patients reported a median of 13 symptoms (IQR = 8–17) and a symptom severity of 35 (IQR = 19–53) (Table 2). The most common symptoms endorsed at initial visit included headache (*n* = 406, 77.0%), difficulty concentrating (*n* = 360, 68.3%), and light sensitivity (*n* = 360, 68.3%). At first follow-up, patients who had completed the VV-HEP reported significantly lower symptom endorsement (median = 1, IQR = 0–3) relative to patients in progress (median = 8, IQR = 3–14; *p* < 0.001) and to patients who had not started (median = 7, IQR = 1–13; *p* < 0.001). Similarly, patients who had completed the VV-HEP reported significantly lower symptom severity (median = 1, IQR = 0–4) relative to patients in progress (median = 15, IQR = 4–30; *p* < 0.001) and to patients who had not started (median = 15.5, IQR = 2–32.5; *p* < 0.001) (Table 3).

**Table 2:** Clinical exam outcomes of all patients at initial visit and first follow-up.

	Initial visit within 28 days	First follow-up within 60 days
**PCSI**, median (IQR)		
* Symptom endorsement*	13 (8–17)	7 (2–13)
* Symptom severity*	35 (19–53)	14 (2–30)
**Visio-vestibular exam**		
Pursuits, *n* (%)	215 (40.8%)	93 (17.7%)
Saccades, *n* (%)		
* Horizontal*	129 (24.5%)	230 (43.6%)
* Vertical*	125 (23.7%)	247 (46.9%)
Vestibular-ocular reflex, *n* (%)		
* Horizontal*	135 (25.6%)	249 (47.3%)
* Vertical*	142 (26.9%)	251 (47.6%)
Near point of convergence, *n* (%)	92 (17.5%)	67 (12.7%)
Accommodation, *n* (%)		
* Left eye*	96 (18.2%)	57 (10.8%)
* Right eye*	83 (15.8%)	49 (9.3%)
Tandem gait, *n* (%)	238 (45.2%)	113 (21.4%)
Total VVE score, *n* (%)	320 (65.2%)	266 (53.4%)

Post-concussion symptom inventory (PCSI) scores reported as median (IQR) and VVE subtests reported as the percentage of patients with an abnormal test.

**Table 3:** Symptom endorsement and severity of patients at follow-up by self-reported progress with the home visio-vestibular program and *post-hoc* pairwise comparisons.

Post-concussion symptom inventory	Has not started	In progress	Completed	Overall *p* value^a^	Effect size (η^2^)	Pairwise comparisons		
						*p*-values^b^		
						Has not started vs. completed	In progress vs. completed	Has not started vs. in progress
Symptom endorsement, median (IQR)	7 (1–13)	8 (3–14)	1 (0–3)	**<0.001**	0.05	**<0.001**	**<0.001**	0.44
Symptom severity, median (IQR)	15.5 (2–32.5)	15 (4–30)	1 (0–4)	**<0.001**	0.04	**<0.001**	**<0.001**	0.91

Bold values indicate a significant *p*-value.

^a^ *p*-value based on Kruskal Wallis test (*α *= 0.05).

^b^ *p*-value based on *post-hoc* pairwise Mann Whitney *U* tests (*α *= 0.017).

At initial visit, 65.2% of patients reported with two or more abnormal VVE subtests and 53.4% of patients reported with two or more abnormal VVE subtests at follow-up (Table 2). When assessing VVE abnormalities by self-reported progress with the VV-HEP at follow-up, we found that a significantly lower proportion of patients who had completed the program reported with abnormal VOR, tandem gait, and total VVE score relative to patients who had not started the program (*p* < 0.005) (Table 4). Additionally, a significantly lower proportion of patients who had completed the VV-HEP reported with abnormal tandem gait and total VVE score relative to patients who were in progress (*p* < 0.005) (Table 4).

**Table 4:** Percentage of patients with abnormal VVE at follow-up by self-reported progress with the home visio-vestibular program and *post-hoc* pairwise comparisons (*α *= 0.005).

	Has not started	In progress	Completed	Overall *p*-value^a^	Effect size (Cramer's V)	Pairwise comparisons		
						*p*-values^a^								
	*n* (%)	*n* (%)	*n* (%)			Has not started vs. completed	In progress vs. completed	Has not started vs. in progress
Pursuits	10 (12.3%)	81 (19.7%)	2 (7.4%)	0.1	0.09	0.47	0.11	0.14
Saccades								
* Horizontal*	39 (48.1%)	182 (44.2%)	6 (22.2%)	0.02	0.12	0.006	0.02	0.21
* Vertical*	40 (49.4%)	198 (48.1%)	6 (22.2%)	0.02	0.13	0.006	0.008	0.53
Vestibular-ocular reflex								
* Horizontal*	42 (51.9%)	198 (48.1%)	6 (22.2%)	0.01	0.14	**0.003**	0.007	0.25
* Vertical*	44 (54.3%)	198 (48.1%)	6 (22.2%)	0.007	0.14	**0.002**	0.007	0.17
Near point of convergence	12 (14.8%)	55 (13.3%)	0	0.1	0.10	0.03	0.04	0.61
Accommodation								
* Left eye*	8 (9.9%)	46 (11.2%)	2 (7.4%)	0.63	0.04	0.91	0.75	0.43
* Right eye*	9 (11.1%)	40 (9.7%)	0	0.24	0.07	0.10	0.17	0.95
Tandem gait	17 (21.0%)	96 (23.3%)	0	0.015	0.13	**0.0045**	**0.004**	0.88
Total VVE score	40 (49.4%)	217 (52.7%)	6 (22.2%)	**0.004**	0.15	**0.003**	**0.0009**	0.88

Bold values indicate a significant *p*-value.

^a^ *p*-value based on chi-square tests (*α *= 0.005).

## Discussion

The purpose of our study was to utilize a large clinical concussion registry to examine the effects of a VV-HEP on symptom reporting and visio-vestibular function during follow-up in concussed pediatric patients. Though a small proportion of patients had completed the VV-HEP within 60 days of injury, our findings indicate that those who did reported with lower symptom endorsement and severity as well as improved visio-vestibular function relative to patients who did not complete the VVE-HEP.

Active and early rehabilitation strategies, including vestibular therapy, have been recommended in recent literature to improve concussion outcomes (3, 6, 7, 29). Previous literature assessing aerobic exercise in adolescent patients have demonstrated faster recoveries following concussion (30) and reduced symptoms (31) and improved mood-related symptoms (32) in patients with persisting symptoms. Similarly, studies of visual and vestibular rehabilitation have found reductions in overall concussion symptoms (18) and reduced recovery times (20). However, some patients experience barriers to access these therapies throughout their full course of care. Mohammed and colleagues (14) found that disparities are evident when examining adherence to concussion care recommendations, some of which may be addressed by using a cost-effective and equitable at-home program. Our findings indicate at-home exercise programs prescribed within 28 days of injury may be effective in reducing overall concussion symptoms. Median symptom endorsement was reduced from 13 symptoms at initial visit to 1 symptom and median symptom severity was reduced from 33 at initial visit to 1 at follow-up in patients who completed the VV-HEP. Similarly, Storey et al. (18). found a reduction from a median of 9 symptoms to 0 symptoms after 2 in-office physical therapy visits and that patients who completed in-office vestibular physical therapy (median = 7 visits) had significantly lower overall symptom burden relative to those who did not complete. The current study indicates that a VV-HEP may be comparably effective to in-office therapy in reducing overall concussion symptoms. Additionally, our study found that patients who reported being in progress with the VV-HEP demonstrated lower median symptom endorsement and severity relative to initial presentation which suggests that the prescription of a VV-HEP does not exacerbate symptoms.

When assessing the effect of the VV-HEP on visio-vestibular function, we found that a lower proportion of patients who had completed the program reported with abnormal aspects of the VVE (horizontal and vertical VOR, tandem gait, total VVE score) relative to patients who were in progress and patients who had not started. Previous studies assessing both in-office and at-home vestibular rehabilitation programs in pediatric patients have demonstrated improvements in visio-vestibular function (18, 19, 22). Alsalaheen et al. (19) found that in-office vestibular therapy supplemented with home exercises significantly improved all elements of the Vestibular/Oculomotor Screening (VOMS) assessment. Our results more closely align with a previous study assessing an at-home program only, which found that a 4-week at-home vestibular rehabilitation program significantly improved horizontal and vertical VOR in concussed patients aged 12–18 (22), suggesting that visio-vestibular function can be improved with home exercises. The additional improvements of tandem gait and total VVE score found in our study can be attributed to the use of different visio-vestibular assessments. The VVE shares domains with the VOMS (smooth pursuits, horizontal and vertical saccades, horizontal and vertical VOR, convergence) with additional exam elements of monocular accommodation and complex tandem gait for a more comprehensive assessment of the visual and vestibular systems (25, 26). Using a similar assessment, Storey et al. (18) found that a larger proportion of patients who did not complete in-office vestibular therapy had abnormal tandem gait performance relative to those who completed therapy. Our findings build on this, suggesting that an at-home program targeting visio-vestibular systems may also be beneficial in improving tandem gait performance.

Our study is not without limitations. Our patients were seen by physicians in a specialty care concussion clinic and therefore, our results may not be generalizable to patients presenting to other health care providers or clinical settings. We were unable to account for additional potential confounding factors between initial visit and follow-up visit such as physical activity, school participation, etc. We excluded patients with missing or invalid date of injury, which is likely not missing at random and reduces the generalizability of the results. Additionally, we relied on patients to self-report their compliance with the VV-HEP which is subject to recall bias.

In summary, we found that patients who reported completing a VV-HEP at first follow-up within 60 days of injury had significantly lower symptom endorsement and severity relative to patients who had not started the program or were in progress. Additionally, a lower proportion of patients who had completed the VV-HEP demonstrated abnormal VOR (horizontal and vertical), tandem gait, and total VVE score. Our findings indicate that an at-home program targeting vision and vestibular function is not only safe but may also be effective in improving clinical outcomes following concussion in pediatric patients. Future research should continue investigating the utilization, compliance, and effectiveness of at-home therapy programs following concussion in pediatric patients in order to improve the accessibility of early and active concussion treatment interventions.

## Data Availability

The datasets presented in this article are not readily available because data were queried from electronic health records of active patients. Requests to access the datasets should be directed to arbogast@chop.edu.
