# Supplementary material for: The effect of a home exercise program on visio-vestibular function in concussed pediatric patients
Source: Front Sports Act Living. 2023 Mar 3;5:1064771. doi: 10.3389/fspor.2023.1064771 (PMC10020172; doi:10.3389/fspor.2023.1064771)
Supplement: Supplementary file 1 [file Table1.docx]

Supplementary Table 1. Visio-vestibular examination (VVE) elements

| Exam element | | Description | Abnormalities |  |
| --- | --- | --- | --- | --- |
| 1 | Smooth Pursuit | Patient follows providers finger horizontally for 5 repetitions | Symptom provocation, jerky or jumpy eye movement, or > 3 beats of nystagmus |  |
|  |  |  |  |  |
|  |  |  |  |  |
| 2,3 | Saccades - horizontal and vertical | Patient looks back and forth with jump eye movements between examiner's fingers (horizontally and vertically) | Symptom provocation at ≤ 20 repetitions |  |
| 4,5 | Vestibulo-ocular Reflex – horizontal and vertical | Patient nods head “yes” or shakes head “no” while fixing eyes on provider’s finger | Symptom provocation at ≤ 20 repetitions |  |
| 6 | Near-Point of Convergence | Patient identifies the distance where 20/20 print letters become double | Doubling of letters >6 cm from patient’s forehead |  |
| 7,8 | Accommodation –  left and right | Patient identifies the distance where 20/20 print letters become blurry | Age-based cut-off determined by Hofstetter’s formula |  |
| 9 | Complex Tandem Gait | Patient walks forward and backward, heel-to-toe, with eyes opened and closed for 5 steps each | >5 errors (steps off straight line) and sway (raising of arms or truncal movement) across all complex tandem gait conditions |  |
|  | Total VVE Score | Overall assessment of previous 9 subtests | ≥2 abnormal subtests |  |
