# Supplementary material for: The effect of a home exercise program on visio-vestibular function in concussed pediatric patients
Source: Front Sports Act Living. 2023 Mar 3;5:1064771. doi: 10.3389/fspor.2023.1064771 (PMC10020172; doi:10.3389/fspor.2023.1064771)
Supplement: Supplementary file 2 [file Datasheet1.pdf]

Vestibular Therapy Home Exercise Program

Complete daily Home Exercise Program for Vestibular Exercises: 1-2 times daily

Work up to 30 repetitions, advancing by 2-3 reps every 2-3 days to 30, 60, then 90 and then 120 reps. Continue until you develop symptoms then do a few more reps to build stamina.

1) **Saccades:**
    a. Horizontal: Put 2 post-it notes on the wall shoulder width apart. Look back and forth right to left for the above recommended repetitions.
    b. Vertical: Place the 2 post-it notes vertical on wall about 12 inches apart and look up and down for the above recommended repetitions.

2) **Gaze Stability:** Put 1 post-it note on the wall in the middle of the 4 post-it notes above.
    a. Horizontal: Keep eyes focused on the post-it note in the middle and nod up and down for the above recommended repetitions.
    b. Vertical: Shake head left and right while focusing on the post-it note in the middle for the above recommended repetitions.

3) **Convergence exercises**
    a. Beads on a String/Brock String: Put 3 beads on a string. One on the end, one in the middle and one as close to you as possible but still in focus (clear). Start by looking at farthest one and focus on each one, getting closer to your nose. Each bead should be in focus (clear, not blurry). As your eyes get better with this exercise, you should be able to move the closest bead closer to your nose, with the goal being 4 cm away from your nose, while it still being clear.
    b. Pencil pushups: Using a pen with letters, start arm's length away and keep in focus as you bring it towards your nose. Once it gets blurry continue slowly until the letters become double - try to keep them single. When they remain double, slowly go in reverse back out to arm’s length. Repeat for about one minute.

4) **Balance:** There are many options for improving balance:
    a. Stand on 1 leg while brushing teeth - hold for 30 seconds.
    b. Wii Balance.
    c. Walk a straight line about 10 feet long - forward, backward with eyes open and eyes closed.
